# Iterative screening methodology enables isolation of strains with improved properties for a FACS-based screen and increased L-DOPA production

**DOI:** 10.1038/s41598-019-41759-0

**Published:** 2019-04-09

**Authors:** Judy Savitskaya, Ryan J. Protzko, Francesca-Zhoufan Li, Adam P. Arkin, John E. Dueber

**Affiliations:** 1 University of California, Berkeley – UCSF Graduate Program in Bioengineering, Berkeley, CA 94720 USA; 2 Department of Bioengineering, University of California, Berkeley, Berkeley, CA 94720 USA; 3 Department of Molecular and Cell Biology, University of California, Berkeley, Berkeley, CA 94720 USA; 4 Environmental Genomics & System Biology, Lawrence Berkeley National Lab, Berkeley, California, USA; 5 Biological Systems & Engineering Division, Lawrence Berkeley National Laboratory, Berkeley, CA 94720 USA

## Abstract

Optimizing microbial hosts for the large-scale production of valuable metabolites often requires multiple mutations and modifications to the host’s genome. We describe a three-round screen for increased L-DOPA production in *S. cerevisiae* using FACS enrichment of an enzyme-coupled biosensor for L-DOPA. Multiple rounds of screening were enabled by a single build of a barcoded *in vitro* transposon-mediated disruption library. New background strains for screening were built for each iteration using results from previous iterations. The same *in vitro* transposon-mediated disruption library was integrated by homologous recombination into new background strains in each round of screening. Compared with creating new transposon insertions in each round, this method takes less time and saves the cost of additional sequencing to characterize transposon insertion sites. In the first two rounds of screening, we identified deletions that improved biosensor compartmentalization and, consequently, improved our ability to screen for L-DOPA production. In a final round, we discovered that deletion of heme oxygenase (HMX1) increases total heme concentration and increases L-DOPA production, using dopamine measurement as a proxy. We further demonstrated that deleting HMX1 may represent a general strategy for P450 function improvement by improving activity of a second P450 enzyme, BM3, which performs a distinct reaction.

## Introduction

Metabolic engineering studies aim to increase production of valuable molecules using microbial hosts. A common strategy is to create a library of DNA mutations and use a screen or selection to isolate library members with increased production. These mutagenesis libraries fall into two categories: on-pathway enzyme mutagenesis and host genome mutagenesis. While several methods for deletion library generation exist, few can be used for successive rounds of screening to identify multiple, complementary mutations without rebuilding the deletion libraries at the beginning of each round. The yeast deletion collection, which comprises a set of *Saccharomyces cerevisiae* strains in which a single ORF has been deleted, has been a major resource for studying genomic deletions conferring desired phenotypes in a barcoded, trackable fashion^1,2^. However, the technical difficulty in recreating deletion libraries in different background strains preclude groups from performing iterative rounds of screening for deletions.

A recent study demonstrated a method called CHAnGE, which can be used to make genomic mutations for iterative mutagenesis and screening. The strategy uses plasmids containing sgRNA and donor DNA sequences to produce Cas9-mediated deletions at specified genomic positions^3^. Another strategy for creating gene knockout libraries in new background strains is to use transposon-disruption^4,5^. To perform multiple iterations of library generation with transposons, each round would require the researcher to generate a new strain library and characterize the insertion sites of the transposons. Furthermore, these insertion sites will be different in each library, making cross-strain comparisons challenging.

We created a barcoded transposon-mediated gene disruption library in an *in vitro* yeast genomic DNA preparation. Using Randomly Barcoded Transposon Sequencing (RB-TNSEQ), barcodes in each transposon can be uniquely mapped to the genomic integration site of the transposon^6^. The key advantage of this strategy is that the transposon-disrupted genome fragment library needs to be generated once and the barcode-insertion position associations only need to be characterized once. The same library can be integrated with high efficiency into any *S. cerevisiae* strain background by homologous recombination. Insertional mutations enriched through selection or screening can then be rapidly identified by PCR amplification and sequencing the associated barcode (BarSEQ). This enables iteration to identify a multi-insertion strain with improved performance.

We demonstrate the value of iterative screening by application to the optimization of heterologous L-DOPA production, a molecule that is often prescribed to treat Parkinson’s disease and an early intermediate for the family of valued benzylisoquinoline alkaloids (BIAs)^7,8^. L-DOPA can be produced by the catalysis of tyrosine hydroxylation by CYP76AD1^9^. Using our iterative method for introducing transposon insertion mutations, we were able to identify mutations that improve our single-cell, fluorescence-activated cell sorting (FACS) screening methodology in the first and second rounds, and a mutation that increases L-DOPA production in our third round.

The pathway to the production of benzylisoquinoline alkaloids (BIAs), which comprise a number of medicinal molecules including morphine, codeine, and noscapine, has garnered considerable attention in yeast metabolic engineering communities owing to the identification and heterologous expression of key genes in its biosynthetic pathway^7,9,10^. BIA production pathways from native plant hosts have been reconstituted in microbial hosts using genes from a variety of organismal sources. Some studies have optimized microbial hosts for the heterologous expression of these pathways^11,12^. However, mutations tested in these studies have been limited to rationally selected pathway-adjacent modifications, rather than genome-wide mutations identified through a screen or selection.

Endogenous pathways for producing BIAs include several cytochrome P450 enzymes (P450s), which are important in many other biosynthetic pathways due to their ability to catalyze the difficult chemistry of forming a new carbon-oxygen bond^7,13–21^. Many efforts to express P450s in fermentative host organisms have been successful, but challenged by difficulties in the expression, folding, cofactor usage, and efficient reduction for catalytic turnover in their non-native hosts^13^. P450 enzymes require a reductase partner that can pass electrons from NAD(P)H cofactors. They also must be loaded with an iron-containing heme group. Natively, P450s are anchored in the endoplasmic reticulum via a transmembrane domain. Each of these factors contribute to the challenge of producing high-titer P450 products in microbial hosts, especially when the biosynthetic pathways contain multiple P450 enzymes.

Previous studies have used protein engineering and directed evolution to modify P450 performance through altered substrate specificity, improved P450-NAD(P)H coupling efficiency, increased tolerance to new reaction conditions, and even altered chemical catalysis^14,22–25^. However, few studies have investigated the optimization of the cellular environment in which heterologously expressed P450s are expressed. Targeting modifications to the host genome may be a promising strategy for improving P450 function since these enzymes use host resources for expression, folding, and cofactor loading.

In this study, we designed a screen of genome-wide deletion libraries in yeast to identify mutants with increased heterologous production of L-DOPA either through availability of the reaction substrate, tyrosine, or improved P450 enzyme activity. Deloache *et al*. recently described an enzyme-coupled biosensor for L-DOPA. The biosensor comprises the expression of DOPA dioxygenase (DOD) from *Mirabilis jalapa*, which converts L-DOPA into betalamic acid. Betalamic acid subsequently condenses with metabolites with a free amine group, such as amino acids, to form fluorescent and colorimetrically detectable betaxanthins^9^. The betaxanthins are able to cross the plasma membrane by an unknown mechanism, complicating single-cell screening methodologies like FACS. We use multiple iterations of mutagenesis and screening to first identify gene deletions that improved the quality of our screen, and then identify mutations that increase product production.

## Results

### Initial screen library construction

For the first round of screening we integrated genes comprising the betaxanthin biosensor into a pooled culture of all strains arrayed in the yeast deletion collection^1^. Barcode sequencing (BarSEQ) demonstrated acceptable uniformity of coverage for the 4,785 unique ORF deletions reported to be present in the deletion collection (Supplementary Fig. S1)^26^. Approximately 88% (4225/4785) of ORFs were represented by more than three reads in pooled sequencing (SI File 1). The deletion collection barcode sequences are known to contain errors^27^. UPtag and DNtag barcodes as well as revised barcode sequences from Eason *et al*. were included in analysis, but 541 ORF deletions still could not be identified by barcode sequencing. Approximately 44% of these missing ORFs (240/541) were found by Eason *et al*. to have mutations in sequencing primer annealing sites, making them inaccessible in pooled sequencing reactions^27^. Transformation of this pooled library with the betaxanthin biosensor cassette yielded approximately 8 × 10^5^ transformants. BarSEQ analysis demonstrated that approximately 85% of all deletion mutants were still represented after transformation.

### Screen of deletion collection mutants for increased betaxanthin

**Figure 1:**
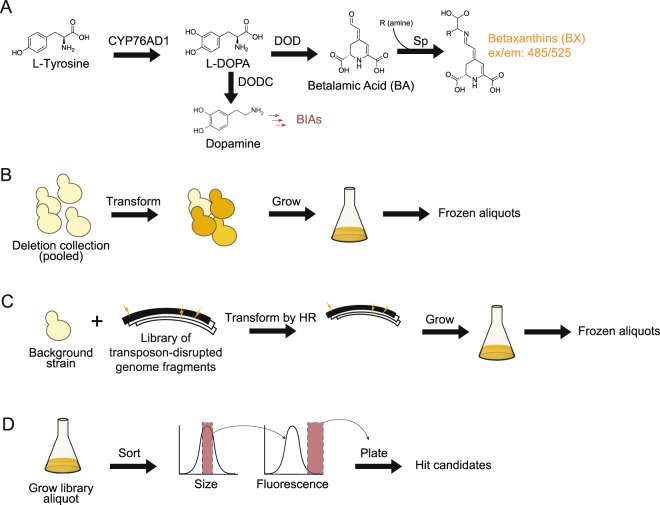
Screening strategy. *In vitro* transposon-disruption library enables iterative screening for deletions that increase production of betaxanthin biosensor. (**A**) The biosensor gene cassette comprises 3 genes: tyrosine hydroxylase (CYP76AD1), DOPA dioxygenase (DOD), as well as an additional feedback-resistant mutant of DAHP synthase (ARO4 K229L); not shown. (**B**) The initial screen was performed using a pooled, transformed culture of the yeast deletion collection. (**C**) Subsequent screen iterations were performed using libraries generated through transposon-based shuttle mutagenesis. Details of cloning for transposon are in Supplementary Fig. S3. (**D**) Libraries generated using the protocols outlined in (**B,C**) were sorted first for size to control for the effects of size on fluorescence. Cells falling in the middle 30% of the size distribution were sorted for fluorescence at a threshold of 0.5% of the population. Sorted cells were plated and hits were later validated and sequenced.

Pooled transformants were screened for high betaxanthin production using fluorescence-activated cell sorting (FACS), gated for the top 0.5% of events. Individual sorted strains were grown on solid agar media (Fig. 1). Out of approximately 1000 colonies, 30 were chosen by visual screening of colonies for high fluorescence and sequenced. Sequencing revealed that of these 30 colonies, 17 harbored a deletion of PDR8, a transcriptional activator of ATP-binding cassette (ABC) transporters and other drug resistance genes. Two of the 30 strains harbored a deletion of QDR2 and one of the 30 strains had IMA5 deleted. The remaining 10 colonies each contained a unique deletion and none of these deletions conferred increased fluorescence when the colonies were picked into liquid media and bulk fluorescence tested, suggesting they were false positives.

**Figure 2:**
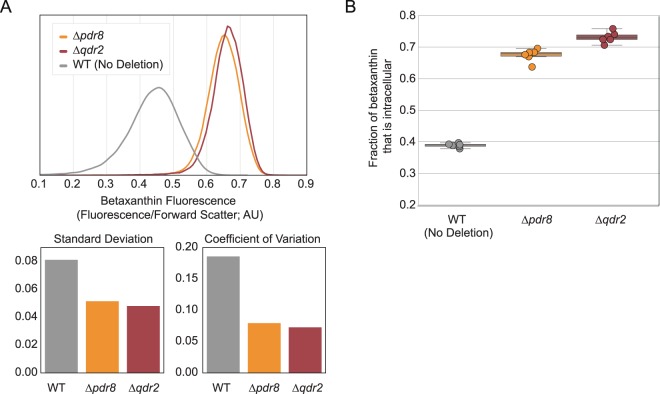
Initial screening identified deletions of PDR8 and QDR2 increase betaxanthin retention. Initial screening identified PDR8 and QDR2 as deletions that increase betaxanthin retention. (**A**) Deletions of these two genes increased single cell fluorescence and decreased the variance in fluorescence measurements within an isoclonal population. The population standard deviation and coefficient of variation are shown below. The resulting coefficient of variation for the *Δpdr8* and *Δqdr2* strains was lower than the WT strain. (**B**) In liquid media, intracellular betaxanthin retention was higher for *Δpdr8* and *Δqdr2* compared to WT. All strains include the same biosensor cassette. Six samples were measured; boxes represent the interquartile range (IQR; 25–75%). Whiskers represent the range of the data with the exception of outliers. Data points outside of the IQR +/− 1.5xIQR are considered outliers.

QDR2 (YIL121W) and IMA5 (YJL216C) are regulatory targets of PDR8^28^. QDR2 is a multidrug resistance transporter protein of the major facilitator superfamily^29^. It is a drug:H+ antiporter (DHA family) and has been shown to export cations^30^. IMA5 is a α-glucosidase known to hydrolyze isomaltose^31,32^. To confirm the phenotypes of these gene deletions, we built markerless knockouts of PDR8, QDR2, and IMA5 in yJS1051, a strain containing the biosensor genes^33^. In these strains, liquid culture measurements of intra- versus extracellular fluorescence showed that deleting QDR2 considerably increased retention of betaxanthin inside the cell and accounted completely for the effect from the PDR8 deletion (Fig. 2). The reason for IMA5 enrichment in the original screen is unclear, since its deletion alone did not increase fluorescence or betaxanthin retention (Supplementary Fig. S2). Given that wildtype strains have high rates of betaxanthin export, we hypothesized that betaxanthin sharing between nearby colonies on an agar plate could increase their perceived betaxanthin production and explain the 11 false positives found in this screen.

### Compartmentalization of betaxanthin improves FACS based detection of L-DOPA

FACS-based screening uses fluorescence of individual cells, so the sharing of fluorescent molecules through secretion into the media can produce false positives. We hypothesized that intracellular compartmentalization of betaxanthin could improve library screening using the betaxanthin biosensor by reducing the betaxanthin transfer between strains. Since the deletion of QDR2 decreased the coefficient of variation in the betaxanthin signal (Fig. 1A), we deleted this gene in our background strain to reduce the frequency of false positives enriched in our FACS screens. Furthermore, we chose to proceed with *Δqdr2*, rather than *Δpdr8*, because we predict its effects to be less pleitropic: PDR8 is known to regulate a number of other genes^28^.

### Second round of host mutagenesis and screening identifies *Δyor1*

After identifying the *Δqdr2* deletion as a host for screening with the L-DOPA biosensor, we sought to create a mutant library within this background strain for discovery of additional host modifications for optimized production of L-DOPA. We created a transposon-mediated gene disruption library in an *in vitro* preparation of yeast genomic DNA from the characterized yeast genome tiling collection^34^. This library could then be transferred into any strain background by homologous recombination with the strain’s genome. Critically, this strategy would enable us to perform multiple rounds of mutagenesis and screening using the same library preparation but update the background strain with each round. In our single library preparation, we sequenced the transposon insertion sites using RB-TnSEQ^6^. For each gene in the genome, we considered a transposon insertion event to be a disruption in a transcribed feature (either an ORF or RNA) if it fell within the first 75% of the feature or within 500 bp upstream of the feature. These heuristics were used to account for the promoter region preceding the gene and the fact that some genes can still be functional even with a 3′ end truncation^6,35^. In this preparation, 58% (4202/7198) of transcribed features had at least one transposition event satisfying these heuristics (SI File 2).

We linearized and integrated this transposon-mediated disruption library into a strain containing the betaxanthin biosensor genes and *Δqdr2* (yJS1159) by transformation and native homologous recombination. We used FACS to identify mutants within this library with higher fluorescence. Four of six isolated mutants contained transposon disruptions of the transporter gene YOR1. We were unable to identify the other two mutants due to poor sequencing data quality.

**Figure 3:**
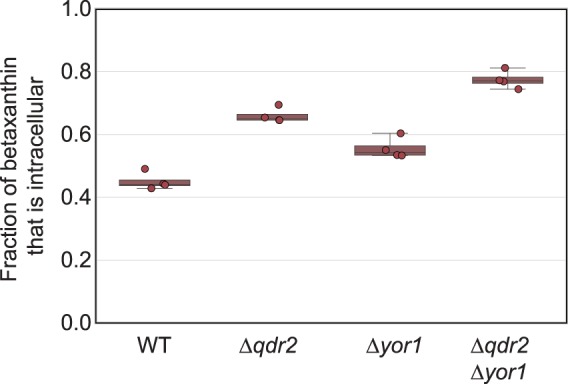
Iterative screening using a transposon-mediated disruption library in the *Δqdr2* strain and decreased CYP expression identified another betaxanthin transporter: YOR1. A transposon-disruption library in a Δqdr2 background enabled identification of a second betaxanthin export mechanism: YOR1. *Δyor1* strain retained more betaxanthin than wild type. The *Δyor1Δqdr2* double mutant strain retained 77.4 +/− 2.8% of betaxanthin produced intracellularly, compared with 45.1 +/− 2.7% in the wild type strain. All four strains contain the same betaxanthin biosensor gene cassette, but expression of CYP76AD1 was driven by pRPL18B, which is approximately 10-fold weaker than the promoter, pTDH3, used to drive expression of CYP76AD1 in the strains shown in Fig. 2. Boxes represent the interquartile range (IQR; 25–75%). Whiskers represent the range of the data with the exception of outliers. Data points outside of the IQR +/− 1.5xIQR are considered outliers.

YOR1 is an ATP-binding cassette (ABC) transporter that is regulated by PDR8, similar to QDR2^28,36^. We verified the phenotype of this YOR1 disruption by building a markerless, Cas9-mediated deletion of YOR1 in our background strain. After measuring intracellular versus extracellular betaxanthin in liquid cultures of this strain, we hypothesized that deletion of YOR1 further decreases efflux of betaxanthin from the intracellular environment (Fig. 3). Furthermore, we verified that combining the markerless deletions of QDR2 and YOR1 in our background strain had an additive effect in compartmentalizing betaxanthin. In a *Δqdr2 Δyor1* strain, generated in a clean background, approximately 78% of the betaxanthin was retained intracellularly (Fig. 3).

### Third round of mutagenesis and screening identifies *Δhmx1*

**Figure 4:**
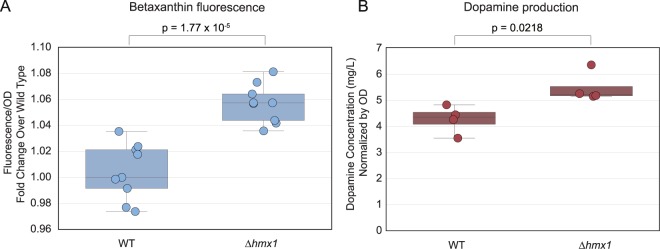
Third iteration of screening identifies *Δhmx1*. Screening of a transposon-disruption library in a *Δqdr2 Δyor1* background led to identification of *Δhmx1*, a disruption of heme oxygenase, that increased both betaxanthin and dopamine titers in their respective production strains. (**A**) The *Δhmx1* strain shows higher total fluorescence than WT in liquid culture. (**B**) The *Δhmx1* strain produces 28.4 +/− 10.5% (s.e.m) more dopamine than WT after a 14 h growth period. Boxes represent the interquartile range (IQR; 25–75%). Whiskers represent the range of the data with the exception of outliers. Data points outside of the IQR +/− 1.5xIQR are considered outliers. Significance was determined using t-tests.

With a *Δqdr2 Δyor1* strain background, we hypothesized that we would be able to uncover beneficial disruption mutants that increase betaxanthin production but were difficult to isolate in previous rounds since they were exporting betaxanthin. In this third round of screening, we integrated the transposon-mediated gene disruption library into a *Δqdr2 Δyor1* biosensor strain (yJS1256) and discovered that the disruption of heme oxygenase HMX1 increased betaxanthin production by approximately 5.2 +/− 0.9% (s.e.m; Fig. 4A). To verify this disruption, we generated a markerless Cas9-mediated deletion of HMX1 in our BY4741 background strain with CYP76AD1 W13L F309L. Measuring L-DOPA is precluded by L-DOPA’s instability during culture extraction and mass spectroscopy. However, its decarboxylated product, dopamine, which is far more stable, can be more easily extracted from spent media. We added the gene DOPA decarboxylase (DODC) to measure dopamine production in these strains and found a 28.4 +/− 10.5% (s.e.m) increase in dopamine production with *Δhmx1*.

### HMX1 may represent a general strategy for P450 function improvement

**Figure 5:**
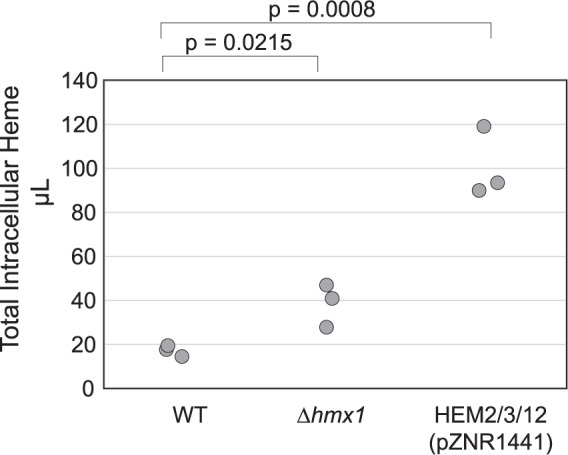
*Δhmx1* increased intracellular heme concentration. The deletion of heme oxygenase HMX1 increases the total intracellular heme concentration by 2.2 +/− 0.4-fold (s.e.m). A previously published strategy of overexpressing heme biosynthesis genes HEM2, HEM3, and HEM12 shows a larger increase in intracellular heme concentration of 5.9 +/− 0.7-fold (s.e.m). Significance was determined using t-tests.

HMX1 encodes heme oxygenase, which is involved in the degradation of heme and regulation of several antioxidant-defense enzymes^37^. We found that deletion of HMX1 increases heme concentration in wild type yeast cells 2.2 +/− 0.4-fold (s.e.m) (Fig. 5). As a comparison and positive control, we also measured the heme concentration in a strain overexpressing heme biosynthesis genes HEM2, HEM3, and HEM12, a known strategy for increasing heme^38^. The overexpression of heme biosynthesis genes has a much larger effect on heme concentrations than the deletion of HMX1.

**Figure 6:**
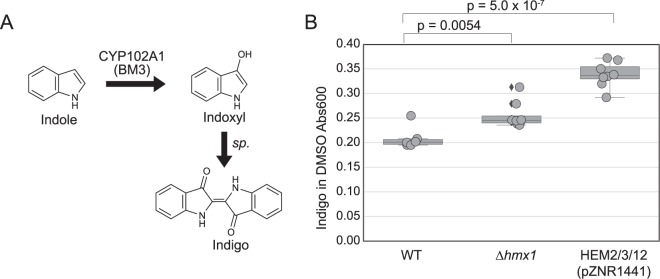
*Δhmx1* also improved activity for a second P450 enzyme. The deletion of heme oxygenase HMX1 improves flux through another P450 enzyme, CYP102A1 (BM3). (**A**) CYP102A1 oxidizes fed indole to indoxyl. Indoxyl spontaneously dimerizes to form indigo, which is dark blue and precipitates out of solution. (**B**) Strains containing CYP102A1 (BM3) produce indigo when fed indole. The *Δhmx1* strain produces approximately 22 +/− 7% (s.e.m) more indigo than the WT strain. Six samples were taken for WT condition and 8 samples were taken for the *Δhmx1* condition. Boxes represent the interquartile range (IQR; 25–75%). Whiskers represent the range of the data with the exception of outliers. Data points outside of the IQR +/− 1.5xIQR are considered outliers and identified with diamonds next to the marker. Significance was determined using a t-test.

We hypothesized that the deletion of HMX1 might be a generalizable strategy for improving the activity of heme-limited P450s. To test this, we integrated CYP102A1 (BM3) into a *Δhmx1* strain. BM3 catalyzes the conversion of indole to indoxyl, which then spontaneously dimerizes to form colored indigo^13,38^. Indigo production increased by 22 +/− 7% (s.e.m) in the *Δhmx1* strain compared with wild type, similar to the improvement observed for CYP76AD1 F309L W13L (Fig. 6). As with the heme production shown in Fig. 5, the previously published strategy of overexpressing HEM2, HEM3, and HEM12 increases indigo production as well and to a greater extent than the deletion of HMX1^38^.

Increases in indigo production through deletion of HMX1 and overexpression of HEM2/3/12 shown in Fig. 6 follow the same trend as increases in heme biosynthesis shown in Fig. 5. This suggests that increased production may be due to alleviation of heme limitation.

## Discussion

In this study, we used iterative host mutagenesis and FACS screening to first identify mutants with improved single-cell screening (*Δqdr2*, *Δyor1*) and, subsequently, a mutant with improved P450 performance (*Δhmx1*). In our initial rounds of screening we discovered that fluorescent product retention is critical for the identification of mutations that increase production because it lowers coefficient of variation for the single cell measurements using this biosensor. We discovered two efflux transporters responsible for exporting approximately 72 +/− 6% (s.e.m) of the betaxanthin produced by the cells. Deletion of these two transporters increased compartmentalization of the fluorescent signal within single cells and enabled screening with a higher signal to noise ratio.

Using the strain with increased betaxanthin compartmentalization (*Δqdr2Δyor1*), a third round of screening led to the discovery that disruption or deletion of heme oxygenase in *S. cerevisiae* leads to increased L-DOPA production. The deletion of heme oxygenase, which is reported to be involved in heme degradation, increases heme levels 2.2 +/− 0.4-fold (s.e.m). Furthermore, addressing heme limitation through deletion of HMX1 or overexpression of heme biosynthesis genes also increased activity of a different P450 enzyme, BM3, which is used in the production of indigo from indole. Taken together, these data suggest that the L-DOPA and indigo pathways tested here may be heme-limited, corroborating previous studies of P450 activity limitations^38,39^.

Importantly, the iterative screening strategy employed in this study was enabled by a barcoded, *in vitro* transposon-based disruption library. Construction and characterization of an *in vitro* library allowed us to perform multiple rounds of screening with updated background strains in each round without needing to rebuild the library for each time. This technique can be used in any organism that can perform homologous recombination with genome fragments from an *in vitro* library with high efficiency.

## Materials and Methods

### Pooling of the deletion collection

The yeast deletion collection was purchased in a format containing 53 96-well plates of strains suspended in glycerol and frozen^1^ (Dharmacon, Lafayette, CO). Plates were thawed and robotic liquid handling (High-throughput sequencing center, UC Berkeley) was used to move 10 μL from each well into a pooling reservoir plate that was kept at 4 °C. The resulting pooled culture was mixed 1:1 with 50% glycerol and was measured to have OD = 62.5. This pooled culture was distributed into 2 mL aliquots and frozen at −80**°**C.

### Strains

All strains were derived from *S. cerevisiae* BY4741. The biosensor cassette was comprised of three genes: (1) *Bv*CYP76AD1 F309L W13L, a cytochrome P450 gene derived from *Beta vulgaris* (beet) containing two mutations for enhanced activity that were identified in a previous publication^9^; (2) *Mj*DOD, dopa dioxygenase derived from *Mirabilis japonica* (4-o’clock flower) that converts L-DOPA to betalamic acid, a precursor to fluorescent betaxanthin; and 3) *Sc*ARO4 K229L, DAHP synthase from the shikimate pathway containing a mutation that inactivates feedback inhibition by tyrosine^40^. After adding the *Δqdr2* mutation following the first screen iteration, the betaxanthin signal was very high. Since we were able to identify improvements from *Δqdr2*, we could ensure our ability to detect further improvements by lowering the betaxanthin signal strength to the levels in the first screen iteration. Thus, strains and libraries built after the first screen iteration contained CYP76AD1 F309L W13L under the control of a weaker promoter than the one used for the first iteration of the screen, pRPL18B (weaker) versus pTDH3 (stronger).

### Media composition

In preparation for DNA transformation, yeast strains were grown in YPD containing 1% yeast extract, 2% peptone, and 2% dextrose (US Biological, Salem, MA). In sorting experiments and fluorescence assays, liquid cultures used synthetic complete media containing 2% dextrose 6.7 g/L yeast nitrogen base with ammonium sulfate (US Biological, Salem, MA). Inositol and amino acids excluding tyrosine and leucine were included at 76 mg/L. Leucine was included at 380 mg/L. Tyrosine was excluded to enable identification of mutants that increase tyrosine production. Adenine was included at 19 mg/L. Uracil was excluded to select for cells successfully transformed with the biosensor genes.

### Preparation of libraries for sorting

Library aliquots were thawed on ice for 30 minutes. The total aliquots were then transferred to 50 mL of synthetic complete media containing 2% dextrose (SD) but lacking uracil and tyrosine. Cultures were grown at 30 °C with shaking for 6 hours. After growth, cultures had an optical density of 9.0–10.0. 8 mL aliquots of library cultures were washed twice with PBS by centrifuging at 2500 × g for 5 minutes and finally resuspended in 8 mL PBS.

### Flow cytometry and library sorting

Library cultures in PBS were sorted using a Sony SH800 Cell Sorter (Sony, Tokyo, Japan). All flow cytometry and sorting were performed using a 488 nm laser and using measurements from a 525/30 band-pass filter. We found a strong correlation between cell size and fluorescence, so we hypothesized that sorting directly based on fluorescence would enrich for mutants that have abnormally large cell size. To account for this, we chose to sort cells that fall into a limited cell size window. This strategy may reduce the probability of enriching for variants with abnormally large or small cell size. However, the population was sorted at mid-log when cells are found at a variety of cell cycle stages and sizes, so abnormally large or small mutants may still fall into the selected size range. Events were first subjected to size-selection by gating for those that fell into the mode of the FSC-A distribution (gate size was set to capture approximately 30% of the population). Cells passing the size-selection gate were then sorted for the top 0.5% of the fluorescence distribution using the excitation and emission listed above. 2000–4000 events were collected and plated on solid media.

### Liquid culture measurements of betaxanthin fluorescence

Liquid cultures were grown in 96 well deep-well blocks for 24 hours. Cultures were back diluted 1:100 into fresh media and aliquoted for fluorescence measurements after 8 hours. Optical density (OD) and fluorescence were measured using a Tecan Spark plate reader (Tecan, Zurich, Switzerland). OD was measured at 600 nm and betaxanthin fluorescence was measured at an excitation/emission of 485/525 nm. Intracellular measurements were performed by aliquoting 100 μL of cell culture into v-bottom 96-well plates and centrifuging the plates at 4700 rpm, then resuspending pellets in 100 μL water before measurement. Extracellular measurements were performed by aliquoting the supernatants from the centrifugation into measurement plates. All measurements were performed using black flat-bottom 96-well plates (Corning, Corning, New York).

### Cas9-mediated markerless gene deletions

Deletions were performed as described in Lee *et al*.^33^. Plasmids were constructed containing Cas9 expression cassettes and sgRNA expression cassettes (pJS1324, pBC909, and pJS1602). Repair DNA was designed as two oligonucleotides 58 bp in length with a 16 bp overlap between them. The remaining 50 bp of each oligonucleotide were homologous to either the sequence immediately upstream of the gene’s start codon or immediately downstream of the gene’s stop codon. Oligonucleotides were transformed along with the Cas9 expression plasmid and the plasmid was selected for by growing on agar media lacking uracil. Selection for the Cas9 plasmid causes cells that have not been edited to suffer toxic double stranded breaks. This serves as a selection for successfully edited cells^33^. After selection of edited cells, Cas9 plasmids were removed by selection with 5-fluoroorotic acid.

### Building the transposon mutagenesis cassette

A schematic of this process is provided in Supplementary Fig. 3. Transposon-based disruption libraries were generated using the transposon shuttle mutagenesis method^4^. A mu transposon was built containing mu repeats flanking a trimR cassette for selection in *E. coli*, a HygR cassette for selection in *S. cerevisiae*, and a 20 bp barcode. The 20 bp randomized barcodes were generated by PCR of the transposon vector with primers GL76 and GL77 which contain degenerate bases at the barcode site. These primers also contain SapI restriction enzyme cut sites that enable ligation of the two ends of the PCR product to form a circular plasmid. Linear DNA fragments containing the barcoded transposons were excised from the vector using BamHI and EcoRI restriction enzyme cut sites flanking the mu repeats and gel purified. *In vitro* transposition was performed by incubating the excised transposon DNA with mu transposase and an existing plasmid library containing genomic fragments^34^. The resulting transposition reaction was transformed by electroporation into *E. coli* TG1 and grown in LB with kanamycin (50 mg/L) and trimethoprim (15 mg/L) to select for plasmids from the genome fragment library that contain successful transposition events (the transposons carry trimR). Plasmid DNA amplified in these cultures was extracted using Zymo Mini Prep Kits (Zymo Research, Irvine, CA). To enrich for plasmids in which the genomic fragments contain the transposition events (as opposed to transpositions in the vector backbone), we used a gateway cloning reaction (Invitrogen) following manufacturer’s protocols to transfer the genomic fragments to a new vector backbone with a different selectable marker (specR). The results of the gateway cloning reaction were transformed by electroporation into *E. coli* TG1 and the plasmid DNA was amplified by growing in LB with trimethoprim (to select for the transposons; 15 mg/L) and spectinomycin (to select for the new backbone without transposition events; 50 mg/L). Plasmid DNA from this culture was extracted using Zymo Mini Prep Kits to obtain the final *in vitro* transposed genome fragment library. In the new backbone, the genomic fragments were flanked by SceI homing endonuclease cut sites, enabling us to excise a linear fragment containing the genomic fragments to use for efficient integrations into the yeast genome by homologous recombination.

### Transposon and barcode sequencing (TnSEQ and BarSEQ)

Illumina-compatible sequencing libraries were generated to link the random DNA barcodes to the transposon insertion sites in the *in vitro* transposed genome fragment library using the method “TnSeq sequencing library preparation” from Wetmore *et al*.^6^, starting with 1ug of the *in vitro* transposed genome library. The resulting library was sequenced on a HiSeq2500 system (Illumina). Barcodes were mapped to the transposon insertion site using a Perl script (MapTnSeq.pl) as described in Wetmore *et al*.^6^. Barcodes in enriched strains were identified by PCR amplification of the barcode region using primers FN53 and FN54 followed by Sanger sequencing.

### Transposon-mediated gene disruption

The *in vitro* transposon library was cut using SceI to create a linear fragment containing transposon insertion sites flanked by yeast genomic DNA regions. Transformation of these fragments was performed following the lithium acetate protocol^41^. Transformants were selected on YPD agar plates supplemented with 300 μg/mL hygromycin. Plates were scraped and resulting cultures were mixed 1:1 with 50% glycerol. 1 mL aliquots of this mixture were stored at −80 **°**C.

### Dopamine measurements

Colonies were picked into 2 mL of synthetic complete medium (minus uracil, tyrosine) with 2% glucose. After overnight growth, saturated cultures were back-diluted 100x into 24-well, deep-well blocks with 2 mL fresh media. The cultures were grown in a Multitron shaker (Infors HT, Bottmingen, Switzerland) for 14 h at 30 °C. Cultures were pelleted, and spent media was removed for further analysis. Dopamine standards were made in spent media of wild type cultures. Samples were mixed 1:1 with *n*-butanol by pipetting up and down 4 times. The organic layer was collected and evaporated, then resuspended in water to prepare for LC/MS.

Samples were loaded onto a Zorbax Eclipse Plus C18 4.6 × 100mm-3.5 μm reversed-phase column (Agilent Technologies, Santa Clara, CA) at ~20 °C using a 0.5 ml/min flow rate. Samples were eluted with a constant mixture of 60% water/40% acetonitrile plus 0.1% formic acid over the course of 20 min. The column was washed with linear gradients up to 100% water and 100% acetonitrile over 5 min. MS was carried out using a 6520 Accurate-Mass Q-TOF LC/MS (Agilent Technologies) for fragmentation and mass detection. The system was run with a fragmentor voltage of 100-V and a collision energy of 23 V. Dopamine ion counts were quantified using m/z of 154.086 [M + H]+ and a retention time of approximately 8.5 min. Dopamine concentrations were quantified against a calibration curve ranging from 0 to 24 mg/L.

### Indigo measurements

Colonies were picked into 0.5 mL of synthetic complete medium (minus uracil) with 2% glucose. Cultures were grown overnight at 30 °C in a Multitron shaker (Infors HT, Bottmingen, Switzerland). Cultures were normalized to OD 0.4/mL using the same media, then 0.1 mL were aliquoted and pelleted. Pellets were resuspended in PBS with 1 mM indole and incubated overnight at 30 °C. Cultures with indigo precipitate were pelleted and resuspended in 100% DMSO to dissolve the indigo. Indigo was quantified at 560 nm absorbance.

### Heme measurements

Total heme was quantified by measuring the fluorescence of protoporphyrin IX (following heme release of iron)^38^. For each sample, 2.4 × 10^8^ cells at OD_600_ = 0.8 were collected, washed with phosphate buffered saline, and resuspended in 500uL of 20 mM oxalic acid in amber tubes. Samples were left at 4 °C for 16 hours in a light-tight box. 500uL 2 M oxalic acid were added to each sample and the sample was split between two tubes, one of which was heated to 98 °C for 30 min. Samples were then centrifuged at 16,000 × g for 2 minutes and supernatants of heated and unheated samples were assayed in a Tecan Spark plate reader (Tecan, Zurich, Switzerland). Measurements were taken at an excitation of 400 nm and emission of 620 nm. Measurements were compared against dilutions of a hemin standard that was treated to the same heating method.

## Supplementary information

Supplementary Information

Supplementary Dataset 1

Supplementary Dataset 2

## Data Availability

All data generated or analyzed during this study are included in this published article (and its Supplementary Information files).
