# Supplementary material for: Iterative screening methodology enables isolation of strains with improved properties for a FACS-based screen and increased L-DOPA production
Source: Sci Rep. 2019 Apr 9;9:5815. doi: 10.1038/s41598-019-41759-0 (PMC6456618; doi:10.1038/s41598-019-41759-0)
Supplement: Supplementary file 1 — Supplementary Information [file 41598_2019_41759_MOESM1_ESM.pdf]

Iterative screening methodology enables isolation of strains with improved properties for a FACS-based screen and increased L-DOPA production

Authors

Judy Savitskaya, Ryan J. Protzko, Francesca-Zhoufan Li, Adam P. Arkin, John E. Dueber*

Supplementary Information

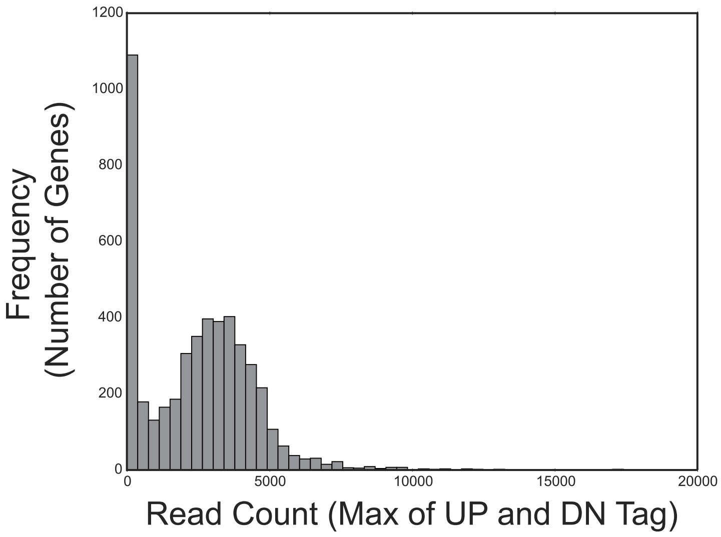

Supplementary Figure S1: Barcode read count distribution for pooled deletion collection. The read counts for 4785 ORFs were binned and plotted as a histogram. For each ORF, the read counts for the UPTAG and DNTAG were both recorded, and the maximum of these two values is shown here. This is to account for mutations or poor sequencing for some barcodes. 510 strains (10.7% of the library) did not have any UPTAG or DNTAG read counts, which is consistent with previous re-sequencing efforts[1]. The median read count is 2,636. A majority of the strains (63.6%) have a read count within a factor of 2 (1,318) or a multiple of 2 (5,272) of the median count.

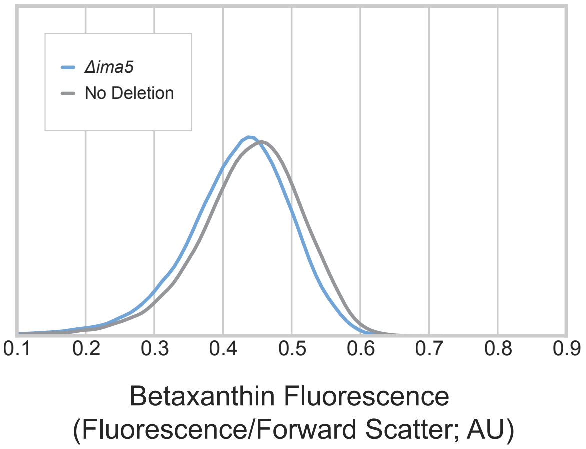

Supplementary Figure S2: *Δima5* did not increase betaxanthin production. *Δima5* was enriched in the first round of sorting. Similar to QDR2, IMA5 encodes a multi-drug family transporter that is regulated by PDR8. However, reconstruction of the IMA5 deletion in a clean background strain shows no improvement in fluorescence over the control. We conclude that IMA5 was a false positive.

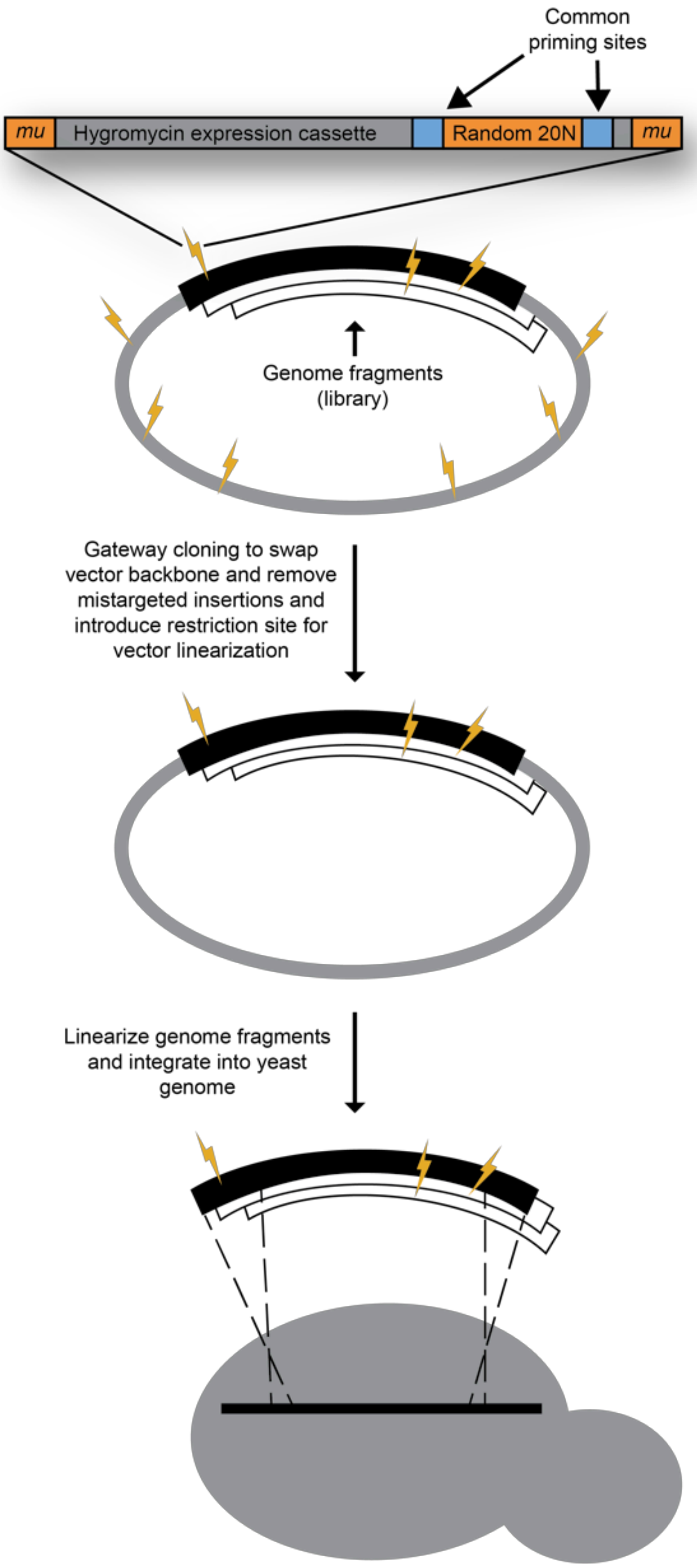
Common
priming sites
mu
Hygromycin expression cassette
Random 20N
mu
Genome fragments
(library)
Gateway cloning to swap
vector backbone and remove
mistargeted insertions and
introduce restriction site for
vector linearization
Linearize genome fragments
and integrate into yeast
genome

Supplementary Figure S3: Construction schematic for the barcoded *in vitro* transposon-based disruption library. Full details are in Materials and Methods. Schematic demonstrates the location of markers, DNA barcode, and priming sites on the transposon. The transposon is integrated into a pool of plasmid vectors containing fragments of the *S. cerevisiae* genome. Gateway cloning is used to move transposon-disrupted genome fragments to a different vector backbone in order to remove transpositions in the backbone and add SceI homing endonuclease recognition sites for linearization. After linearization by SceI, transposon-disrupted genome fragments are integrated into any *S. cerevisiae* strain by transformation and homologous recombination.

Supplementary Dataset 1
We aliquoted cultures of the 4785 unique strains in the deletion collection into a common pool. SupplementaryFile1.csv gives the sequencing counts for each member of the deletion collection in the pool. The “UPTAG_list” and “DNTAG_list” columns contain the barcodes known to represent the ORF from the original publiation of the deletion collection and from the revised barcodes reported by Eason *et al*. who used sequencing to identify mutations in the barcodes. The “UPTAG_counts” column contains the sum of counts for all barcodes in the “UPTAG_list”. Similarly, the “DNTAG_counts” column contains the sum of counts for all barcodes in the “DNTAG_list”.

Supplementary Dataset 2
Transposon insertion sites were identified using RB-TnSEQ[2]. This file lists DNA features (ORFs, expressed RNAs). For each feature, “scaffoldID” identifies the chromosome number, “strand” indicates if the feature is located on the positive or negative strand, “begin” specifies the first base in the feature relative to the chromosome start, and “end” specifies the last base in the feature relative to the chromosome start. All features and sequence information is taken from the Saccharomyces Genome Database S288C sequencing data. The “barcodes” column contains the list of barcodes for transposons that inserted into the genome. The “relLoc” column gives the position of the transposon insertion, relative to the first base in the feature (“begin” if the feature is on the (+) strand, “end” if the feature is on the (-) strand).

Supplementary Table S1: Plasmids
All plasmids were constructed using the yeast toolkit mo-clo system previously described by our lab[3]. All plasmid backbones contained ColE1 replication origins.

Name	Plasmid contents
pJS1051	For integration into S. cerevisiae following linearization by NotI: pTDH3-CYP76AD1_H3H_W13L_F309L-tTDH1-pCCW12-DOD-tADH1-pPGK1-ARO4_K229L-tPGK1; KanR *E. coli* marker; yeast URA3 locus homology arms, flanked by NotI restriction enzyme sites
pJS1475	For integration into S. cerevisiae following linearization by NotI: pRPL18B-CYP76AD1_H3H_W13L_F309L-tTDH1-pCCW12-DOD-

	tADH1-pPGK1-ARO4_K229L-tPGK1; KanR *E. coli* marker; yeast URA3 locus homology arms, flanked by NotI restriction enzyme sites
pPSG533	Spacer sequence; KanR *E. coli* marker; *S. cerevisiae* HIS3 expression cassette; *S. cerevisiae* Cen6 replication origin
pZNR1441	pCCW12-HEM2-tENO2-pTDH3-HEM12-tSSA1-pTEF1-HEM3-tTDH1; KanR *E. coli* marker; *S. cerevisiae* HIS3 expression cassette; *S. cerevisiae* Cen6 replication origin
pJS1324	pPGK1-Cas9-tPGK1-sgRNA targeting QDR2; KanR *E. coli* marker; yeast URA3 expression casette; *S. cerevisiae* Cen6 replication origin
pBC909	pPGK1-Cas9-tPGK1-sgRNA targeting YOR1; KanR *E. coli* marker; yeast URA3 expression casette; *S. cerevisiae* Cen6 replication origin
pJS1602	pPGK1-Cas9-tPGK1-sgRNA targeting HMX1; KanR *E. coli* marker; yeast URA3 expression casette; *S. cerevisiae* Cen6 replication origin

Supplementary Table S2: Strains

All strains were constructed using the base strain BY4741: *MATa his3Δ1 leu2Δ0 met15Δ0 ura3Δ0.* Purchased deletion collection strains were derived from the same background. *In vitro* transposon insertion library was constructed using genomic DNA isolated from this strain.

**Name**	**Genotype**
yJS1051	*ura3::pTDH3-CYP76AD1_H3H_W13L_F309L-tTDH1-pCCW12-DOD-tADH1-pPGK1-ARO4_K229L-tPGK1*
yJS1067	*pdr8Δ0; ura3::pTDH3-CYP76AD1_H3H_W13L_F309L-tTDH1-pCCW12-DOD-tADH1-pPGK1-ARO4_K229L-tPGK1*
yJS1068	*qdr2Δ0; ura3::pTDH3-CYP76AD1_H3H_W13L_F309L-tTDH1-pCCW12-DOD-tADH1-pPGK1-ARO4_K229L-tPGK1*
yJS1159	*qdr2Δ0; ura3::pRPL18B-CYP76AD1_H3H_W13L_F309L-tTDH1-pCCW12-DOD-tADH1-pPGK1-ARO4_K229L-tPGK1*
yJS1256	*yor1Δ0; qdr2Δ0; ura3::pRPL18B-CYP76AD1_H3H_W13L_F309L-tTDH1-pCCW12-DOD-tADH1-pPGK1-ARO4_K229L-tPGK1*
yJS1257	*yor1Δ0; ura3::pRPL18B-CYP76AD1_H3H_W13L_F309L-tTDH1-pCCW12-DOD-tADH1-pPGK1-ARO4_K229L-tPGK1*
yJS1337	*hmx1Δ0; ura3::pRPL18B-CYP76AD1_H3H_W13L_F309L-tTDH1-pCCW12-DOD-tADH1-pPGK1-ARO4_K229L-tPGK1*
yJS1221	*ura3::pTDH3-CYP76AD1_H3H_W13L_F309L-tTDH1-pCCW12-DODC-tADH1-pPGK1-ARO4_K229L-tPGK1*
yJS1343	*hmx1Δ0; ura3::pTDH3-CYP76AD1_H3H_W13L_F309L-tTDH1-pCCW12-DODC-tADH1-pPGK1-ARO4_K229L-tPGK1*
yJS1372	*ura3::pTDH3-CYP102A1_G4-mRuby2-tADH1* plasmid pPSG533
yJS1373	*hmx1Δ0; ura3::pTDH3-CYP102A1_G4-mRuby2-tADH1* plasmid pPSG533
yJS1558	plasmid pPSG533
yJS1559	*hmx1Δ0;* plasmid pPSG533
yJS1560	plasmid pZNR1441

Supplementary Table S3: Oligos

Name	Purpose	Sequence
GL76	Generate barcode	CTTAGCTCTTCACGTACGCTGCAGGTCGAC
GL77	Generate barcode	GAATGCTCTTCAACGNNNNNNNNNNNNNNNNN NNNAGAGACCTCGTGGACATC
FN53	Sequence barcode	AATGATACGGCGACCACCGAGATCTACACTCT TTCCCTACACGACGCTCTTCCGATCTNNNNNG TCGACCTGCAGCGTACG
FN54	Sequence barcode	CAAGCAGAAGACGGCATACGAGATCTGATCGT GACTGGAGTTCAGACGTGTGCTCTTCCGATCT GATGTCCACGAGGTCTCT
HY56	QDR2 repair DNA F	TGCTGGTCATTTTAGTAGAAACTCTGCTCTCAA ACTTGAGTACTGCAACGGAAGTAAA
HY57	QDR2 repair DNA R	GTGGAGCGATCAAAAGGAACATTTTCCTTTGAT TCAAGAAGCTTTACTTCCGTTGCAG
HU80	YOR1 repair DNA F	CGCTAGAATTGAATTTGCCTTATCTTTTCAGCC GTTTTTGGCTGGGTTCCCGCGGCTT
HU81	YOR1 repair DNA R	GACCAGGCAATTGTAATACATAAGTCAACAAAA CACCAACTGAAGCCGCGGGAACCCA
IE66	HMX1 repair DNA F	GcacaatataacacagcatatatacacacacacacataAAATA ACCGCAAAACGTATaaa
IE67	HMX1 repair DNA R	ATATTATTTCATGTATATATTATGTTTGTATTTAG ACttttttttttATACGTTTTGCGG

Supplementary Information References

1. Eason, R. G. *et al.* Characterization of synthetic DNA bar codes in *Saccharomyces cerevisiae* gene-deletion strains. *PNAS* **101,** 11046–11051 (2004).
2. Wetmore, K. M. *et al.* Rapid quantification of mutant fitness in diverse bacteria by sequencing randomly bar-coded transposons. *MBio* **6,** e00306–15 (2015).
3. Lee, M. E., DeLoache, W. C., Cervantes, B. & Dueber, J. E. A Highly Characterized Yeast Toolkit for Modular, Multipart Assembly. *ACS Synth. Biol.* **4,** 975–986 (2015).
